# Identification and Characterization of an Ecto-Pyrophosphatase Activity in Intact Epimastigotes of *Trypanosoma rangeli*

**DOI:** 10.1371/journal.pone.0106852

**Published:** 2014-09-09

**Authors:** André Luiz Fonseca-de-Souza, Anita Leocadio Freitas-Mesquita, Lisvane Paes Vieira, David Majerowicz, Nathalia Daflon-Yunes, Lia Carolina Almeida Soares-de-Medeiros, Kildare Miranda, Katia Calp Gondim, José Roberto Meyer-Fernandes

**Affiliations:** 1 Laboratório de Terapia e Fisiologia Celular e Molecular, Centro Universitário Estadual da Zona Oeste, Rio de Janeiro, RJ, Brazil; 2 Institutode Bioquímica Médica Leopoldo de Meis, CCS, Universidade Federal do Rio de Janeiro, Rio de Janeiro, RJ, Brazil; 3 Instituto Nacional de Ciência e Tecnologia em Biologia Estrutural e Bioimagem, Rio de Janeiro, RJ, Brazil; 4 Instituto de Biofísica Carlos Chagas Filho, CCS, Universidade Federal do Rio de Janeiro, Rio de Janeiro, RJ, Brazil; 5 Instituto Nacional de Ciência e Tecnologia em Entomologia Molecular, Rio de Janeiro, RJ, Brazil; Federal University of São Paulo, Brazil

## Abstract

In this study, we performed the molecular and biochemical characterization of an ecto-enzyme present in *Trypanosoma rangeli* that is involved with the hydrolysis of extracellular inorganic pyrophosphate. PCR analysis identified a putative proton-pyrophosphatase (H^+^-PPase) in the epimastigote forms of *T. rangeli*. This protein was recognized with Western blot and flow cytometry analysis using an antibody against the H^+^-PPase of *Arabidopsis thaliana*. Immunofluorescence microscopy confirmed that this protein is located in the plasma membrane of *T. rangeli*. Biochemical assays revealed that the optimum pH for the ecto-PPase activity was 7.5, as previously demonstrated for other organisms. Sodium fluoride (NaF) and aminomethylenediphosphonate (AMDP) were able to inhibit approximately 75% and 90% of the ecto-PPase activity, respectively. This ecto-PPase activity was stimulated in a dose-dependent manner by MgCl_2_. In the presence of MgCl_2_, this activity was inhibited by millimolar concentrations of CaCl_2_. The ecto-PPase activity of *T. rangeli* decreased with increasing cell proliferation *in vitro*, thereby suggesting a role for this enzyme in the acquisition of inorganic phosphate (Pi). Moreover, this activity was modulated by the extracellular concentration of Pi and increased approximately two-fold when the cells were maintained in culture medium depleted of Pi. All of these results confirmed the occurrence of an ecto-PPase located in the plasma membrane of *T. rangeli* that possibly plays an important role in phosphate metabolism of this protozoan.

## Introduction

In the Trypanosomatidae family, the genus *Trypanosoma* comprises digenetic flagellates that typically have insects as vectors and infect human beings and other animals as hosts. *Trypanosoma rangeli* is a parasite of several wild and domestic animals, with a wide geographical distribution in many Latin American countries, but apparently unable to elicit pathology [1], [2]. Despite being harmless to mammals, *T. rangeli* is considered pathogenic to its insect vectors, being mainly transmitted by triatomine bugs of the genus *Rhodnius* [3]. The basis of the *T. rangeli* infection in *Rhodnius prolixus* remains poorly understood. The parasite and its vector engage in a series of interactions resulting in oral infection; trypomastigotes differentiate into epimastigotes, which multiply in the gut, and the parasite penetrates through the epithelial cells of the digestive tract to enter into the hemocoel, where they freely multiply in the hemolymph or inside the hemocytes. The parasites complete their growth in the salivary glands, the site of metacyclogenesis [2]–[8].

During its life cycle, *T. rangeli* can be subjected to pH variations. Mechanisms to cope with this varied environmental pH and to maintain cytosolic pH homeostasis might involve the use of proton pumps on the plasma membrane and on the internal membranes. These transport proteins specifically and actively mobilize ions (specifically protons), thereby generating chemical gradients across the membrane. This movement of ions is vital for numerous cellular functions, ranging from energy production, motility, nutrients uptake, ionic homeostasis, intracellular signaling and differentiation, among others [9].

Inorganic pyrophosphate (PPi) has been suggested to be the predecessor of ATP in the early stages of evolution and as an alternative “energy currency” in the bioenergetics of certain modern cells [10]. Among various analytes, PPi is a biologically important target because it is the product of ATP hydrolysis under cellular conditions [11]. PPi also plays an important role in energy transduction in organisms and can control metabolic processes by participating in enzymatic reactions [12]. Moreover, PPi has recently been described as an anti-inflammatory molecule [13].

Recent developments have led to a new overall view of the proton-translocating inorganic pyrophosphatases (H^+^-PPases). These unique proton pumps involving PPi and not ATP have presented a novel angle for research on the most elusive coupling mechanism linking anhydrous phosphate energy with active proton transport [14].

H^+^-PPases belong to a recently identified category of proton pumps that are distinct from F-, P- and V-ATPases and utilize pyrophosphate hydrolysis as the driving force for H^+^ movement across biological membranes [15]. In prokaryotic species, H^+^-PPases reside in the plasma membrane, whereas in eukaryotic species, the enzyme is commonly found in the membranes of organelles, such as vacuoles in plants [15] and acidocalcisomes in protozoa [16]. In addition, a previous report has indicated that the H^+^-PPase of the bacterium *Agrobacterium tumefaciens* is located in acidocalcisome-like organelles [17]. The substrate-binding domain faces the cytoplasm, independent of the H^+^-PPase subcellular localization. The PPi-hydrolyzing and proton translocation activities are associated with a single 66-90 kDa polypeptide [18], [19], [20] that possibly forms a dimer [21], [22]. H^+^-PPases have been characterized at the biochemical and genetic levels in various higher plants [23], [24], in a few eubacteria [10], [18], [25], and more recently, in an archaea [26] and in certain human pathogenic protozoa [27]–[35].

H^+^-PPases have been reported to occur in the acidocalcisomal membranes of a number of pathogenic trypanosomatid and apicomplexan protozoa causative of endemic tropical diseases [27], [28], [31], and the genes encoding for these proteins have been cloned and sequenced in a number of parasites, including *Trypanosoma cruzi* [34], *Trypanosoma brucei* [36] and *Toxoplasma gondii* [35]. Plasma membrane-bound PPase activities have been described in *Leishmania donovani* and *Leishmania amazonensis* [9], [37].

H^+^-PPases do not appear to be present in mammals; therefore, they might be potential targets for vaccines and drugs against pathogenic protists, most of them with an intracellular lifestyle. Consequently, studies on the genetics and biochemistry of H^+^-PPases in these organisms might be of significant importance. The presence of genes encoding for H^+^-PPases in the genome of different parasitic and free-living protists of different phylogenetic groups has been further confirmed using Southern blot analyses and PCR-amplified DNA fragments common to the well-known H^+^-PPases of plants and the proteobacterium *Rhodospirilum rubrum* [38].

Either Mg^2+^ or Mn^2+^ can act as a PPase cofactor, and the relative activating efficiency of these cofactors is the key feature that distinguishes family I from family II enzymes. Family I PPases are activated most efficiently by Mg^2+^, whereas their Mn^2+^-supported activity is two orders of magnitude lower [39], [40]. Alternatively, family II PPases prefer Mn^2+^ as a cofactor and are 20-times less active compared with the Mg^2+^-activated [41], [42]. In addition, the H^+^-PPases have recently been divided into two primary types based on their kinetic parameters of activity. Type I H^+^-PPases are moderately sensitive to inhibition by Ca^2+^, whereas type II H^+^-PPases are highly sensitive to Ca^2+^. Both of these types are inhibited by aminomethylenediphosphonate (AMDP), imidodiphosphate (IDP), and high concentrations of sodium fluoride (NaF) and are active only in the presence of Mg^2+^ [43].

Several possible roles have been suggested for PPases in addition to H^+^-translocation and Pi availability. The *in vitro* development and molting of *Ascaris suum* L3 to L4 larvae stages were efficiently inhibited in a dose-dependent manner by imidodiphosphate and sodium fluoride, both of which are potent inhibitors of soluble- and membrane-bound H^+^-PPases [44]. Subsequently, this same group demonstrated that fluoride exposure inhibits the protein expression levels of pyrophosphatase [45]. When considering additional organisms that are closer to *T. rangeli*, it has been shown that the growth of *T. cruzi* [46] and *T. gondii* [33] *in vitro* and *in vivo* is blocked by pyrophosphate analogs and that pyrophosphate is more abundant than ATP in the replicating forms of the parasites.

The immunoelectron microscopy and surface biotinylation data supported the presence of *T. cruzi* H^+^-PPase in the plasma membrane, and the biochemical experiments demonstrated that this enzyme presents unique characteristics compared with those located in acidocalcisomes. The exact localization of these enzymes was unclear (either the external or internal surface of the parasites) because the authors utilized plasma membrane vesicles [47]. The only report of H^+^-PPase activity as an ectoenzyme has been described in Archaea, such as the *Sulfolobus* genus [48], [49]. Considering these findings, in this study, we report an ecto-PPase activity located on the external surface of the trypanosomatid parasite, *T. rangeli*. The presence of this enzyme at the plasma membrane was determined using a rabbit anti-plant-type vacuolar H^+^-PPase antibody and presented biochemical characteristics that resembled internal membrane-bound H^+^-PPases described in several organisms.

In addition, our group demonstrated that *T. rangeli* is a trypanosomatid parasite that strongly depends on the inorganic phosphate (Pi) content for proliferation. This parasite also presents an ecto-phosphatase that responds to Pi starvation in culture medium, thereby suggesting the existence of a Pi-regulating gene system [50], [51]. Therefore, in this study, we demonstrate that ecto-PPase is also positively regulated when Pi is at a low concentration in the culture medium, and these data suggest that *T. rangeli* can hydrolyze PPi, thereby generating the Pi that is required for cellular functions in parasites.

## Materials and Methods

### 1. Parasites and the growth conditions

The Macias strain of *T. rangeli* (supplied by Dr. Maria Auxiliadora de Sousa, from Fiocruz, Rio de Janeiro, Brazil) was maintained in liver infusion tryptose medium (LIT) supplemented with 20% fetal calf serum (Gibco, Life Technologies) at 28±2°C and subcultivated at 5-day intervals in which the parasites achieved a stationary phase of growth [50], [51]. The Pi-supplemented culture medium consisted of sodium chloride 4.0 g/l, potassium chloride 0.4 g/l, disodium hydrogen phosphate 7.1 g/l, glucose 2 g/l, liver infusion broth 5.0 g/l, tryptose 5.0 g/l, hemin 200 mg/l and folic acid 30 mg/l. The pH was adjusted to 7.2 with HCl. In the Pi-starved culture medium, the disodium hydrogen phosphate was replaced with 8.4 g/l sodium bicarbonate. The pH was also adjusted to 7.2 with HCl. The measurements of the Pi concentrations in the Pi-supplemented culture medium (50 mM) and in the Pi-starved culture medium (2 mM) were determined according to the Fiske and Subbarow method [52]. For the experiments, the parasites were harvested from the culture medium using centrifugation at 1500 x *g* at 4°C for 10 min and washed twice in a cold buffer solution containing 100 mM sucrose, 20 mM KCl and 50 mM Tris (pH 7.2). The cellular viability was evaluated before and after the incubations with motility and Trypan blue dye exclusion. Briefly, for Trypan staining, the cells were incubated in the presence of 0.01% Trypan blue for 10 min in the buffer used for each experiment [53]. The cell viability was unaffected under the conditions employed in this study.

### 2. Materials

All of the reagents were obtained from E. Merck (D-6100 Darmstadt, Germany) or Sigma-Aldrich Chemical Company (St. Louis, MO). The distilled water was deionized using a Milli-Q system of resins (Millipore Corp., Bedford, MA) and was used in the preparation of all of the solutions. All of the other reagents were of analytical grade.

### 3. Putative proton-pyrophosphatase gene expression

The amino acid sequence of *Arabidopsis thaliana* vacuolar H^+^-PPase (AVP2) (GenBank access number: NP_001117619) was used as the query sequence in a search against the *T. cruzi* genomic sequence using the TBLASTN algorithm. The best hit was a putative *T. cruzi* vacuolar-type proton translocating pyrophosphatase (GenBank access number: XP_813460), and a pair of primers with the following sequence was designed to amplify part of this mRNA: TcHPPase-1f: 5′ – TTCGCCATGTACTGGTGGTA – 3′ and TcHPPase-1r: 5′ – ACGCACTCGTACACGTTCAG – 3′. To evaluate the expression of a putative pyrophosphatase gene in *T. rangeli*, 10^8^ parasites were homogenized in TRIzol (Life Technologies, Carlsbad, USA), and the total RNA was extracted from the samples according to the manufacturer's instructions. The total RNA concentrations were determined spectrophotometrically using a Nanodrop ND-1000 spectrophotometer (Thermo Fisher Scientific, Wilmington, USA). One microgram of total RNA was treated with 1 U of RNase-free DNaseI (Fermentas International, Burlington, Canada) for 1 hour at 37°C and was then used to synthesize the cDNA samples using the High-Capacity cDNA Reverse Transcription kit (Life Technologies) according to the manufacturer's instructions (2 hours at 37°C in a final volume of 20 µl using random primers). The cDNA samples (0.4 µl in a final volume of 20 µl) were used as the templates in a PCR reaction using Taq DNA Polymerase (Fermentas) with the primers described above at a final concentration of 0.3 µM under the following conditions: 1 cycle for 2 min at 94°C, followed by 30 cycles of 30 s at 94 °C, 30 s at 57°C and 30 s at 72°C, and one cycle for 10 min at 72°C. The PCR products were subjected to agarose gel electrophoresis, stained with ethidium bromide and photographed under a UV light. The amplification of *TrGAPDH* (TrGAPDHf: 5′ – GCAGCTCCATCTACGACTCC – 3′ and TrGAPDHr: 5′ – AGTATCCCCACTCGTTGTCG – 3′) was used as the positive control. The samples derived from the cDNA synthesis reaction without reverse transcriptase were used as the PCR negative control (**Figure S1**).

To confirm the *TrHPPase* amplification, the PCR product band was excised from the agarose gel, and the DNA was purified using the GeneJET Gel Extraction and the DNA Cleanup Micro Kit (Thermo Fisher Scientific). The purified product was sequenced at Laboratório Sonda (Rio de Janeiro, Brazil). The obtained sequence is deposited at GenBank under accession number KM245577.

### 4. Western blot analysis

For the Western blot, the total extracts of the *T. cruzi* and *T. rangeli* epimastigotes (50 µg/lane) were subjected to 8% SDS-polyacrylamide gels [54] and subsequently transferred to a nitrocellulose membrane. The membranes were blocked with 5% (w/v) nonfat dry milk in TBS-T (10 mM Tris-HCl, pH 8.0, 150 mM NaCl containing 0.1% v/v Tween 20) and maintained overnight at 4°C. The membranes were washed three times for 15 min with TBS-T buffer and incubated with the rabbit primary antibody raised against a conserved domain (326 peptide) of AVP2 at a dilution of 1∶1000 for 2 hours at 4°C. After washing with TBS-T, the membranes were incubated with the secondary antibody peroxidase-conjugated goat anti-rabbit diluted to 1∶5000 (Santa Cruz Biotechnology, Inc., Santa Cruz, USA) and were revealed using an ECL PLUS detection kit (Amersham-Pharmacia Biotech, Little Chalfont, UK).

### 5. Fluorescence-activated cell sorter (FACS) analysis

The *T. rangeli* cells (5×10^6^) were prepared using the procedure for obtaining the cell mass as described above. After the final wash, the cell mass was resuspended in a buffer solution of sodium cacodylate 0.5 M, pH 7.4, containing 4% paraformaldehyde. After 30 min of incubation, the cells were centrifuged and washed three times in the same buffer without paraformaldehyde. After the final wash, the cells were resuspended in a phosphate buffered saline (PBS) containing 5% fetal calf serum (FCS) (PBS-FACS) in the absence or presence of a rabbit IgG primary antibody raised against a conserved domain (326 peptide) of AVP2 at a dilution of 1∶150 in a final volume of 100 µL. After a 2 h incubation at 4°C, the cells were centrifuged at 1500 *g* for 2 min and washed three times with PBS-FACS; subsequently, the supernatant was discarded. After the final wash, the cells were resuspended in PBS-FACS in the presence of an Alexa 488-conjugated mouse IgG anti-rabbit antibody at a dilution of 1∶400 in a final volume of 100 µL. After a 2 h incubation in the dark, the cells were centrifuged at 1500 g for 2 min and rinsed twice in PBS-FACS. The cells were resuspended in 400 µL PBS-FACS and analyzed on a FACSCan flow cytometer (Beckton and Dickinson). The cells were separated in a region according to their granularity and size, and in this region, 10,000 cells were acquired for analysis.

### 6. Immunolocalization of H^+^-PPase

The cells fixed in freshly prepared 4% formaldehyde were allowed to adhere to poly(l-lysine)-coated coverslips blocked with 50 mM ammonium chloride and 3% bovine serum albumin in PBS. Immunofluorescence was performed in the cells either permeabilized or non-permeabilized for 1 min with 1% Triton X-100 using a 1∶150 dilution of monoclonal antibody raised against a conserved domain (326 peptide) of AVP2 and an Alexa Fluor-conjugated goat anti-mouse IgG secondary antibody (1∶300). The images were obtained using a Zeiss Axioplan epifluorescence microscope equipped with an Olympus XC30 digital camera [43]. The image deconvolution was performed using a no-neighbor algorithm, as described in the literature [55].

### 7. Ecto-pyrophosphatase activity measurements

The pyrophosphatase activity was determined using sodium pyrophosphate (PPi) as the substrate to measure the rate of Pi production. The epimastigotes of *T. rangeli* (10^7^ cells) were incubated at 25°C for 60 min in 0.6 ml of a reaction mixture containing 100 mM sucrose, 20 mM KCl, 50 mM Tris-HCl, pH 7.2 and 1 mM PPi as a substrate. The reactions were initiated by the addition of the intact cells and were interrupted with the centrifugation of the tubes at 1500 x *g* for 15 min at 4°C. Aliquots (0.5 ml) were transferred to fresh tubes. The Pi release was measured according to the Fiske and Subbarow method [52]. Briefly, released phosphate was added to a solution containing ferrous sulfate 8% (w/v), ammonium molybdate 50% (v/v) and sulfuric acid 20% (v/v) to produce a complex of ammonium phosphomolybdate, which was then measured spectrophotometrically at 650 nm using a Pi curve as the standard. The PPase activity was calculated by subtracting the nonspecific PPi hydrolysis measured in the absence of the parasite cells. In the experiments in which high concentrations of metals were tested, the possibility of precipitate formation was checked as previously described [56]. Under the conditions employed in this study, in the reaction medium containing 100 mM sucrose, 20 mM KCl, 50 mM Tris-HCl (pH 7.2) and 1 mM PPi, no phosphate precipitates were observed in the presence of these cations.

### 8. Measurement of the ecto-phosphatase and ecto-ATPase activities

The ecto-phosphatase activity was determined by measuring the rate of Pi release from β-glycerophosphate (β-GP). The intact epimastigotes of *T. rangeli* (10^7^ cells) were incubated at 25°C for 60 min in the same reaction mixture described in section 7 (Materials and Methods), using 1 mM β-GP as the substrate. The reactions were initiated by the addition of the intact cells and were interrupted by centrifugation of the tubes at 1500 x *g* for 15 min at 4°C. Aliquots (0.5 ml) were transferred to other tubes. The Pi release was measured according to the Fiske and Subbarow method [52], as described above. To determine the ecto-ATPase activity, the intact cells (10^7^ cells) were incubated at 25°C for 60 min in 0.5 ml of a mixture containing the same medium described in section 7 (Materials and Methods), in the presence or the absence of 5 mM MgCl_2_ and using 5 mM ATP as a substrate. The ecto-ATPase activity was calculated from the total activity, which was measured in the presence of MgCl_2_, minus the basal activity, which was measured in the absence of MgCl_2_. This activity was determined by measuring the hydrolysis of [γ^32^P] ATP (10^4^ Bq/nmol ATP) [56]. The experiments were initiated by the addition of living cells and were terminated by the addition of 1.0 ml of a cold mixture containing 25% charcoal in 1.0 M HCl. The tubes were then centrifuged at 1500 x *g* for 15 min at 4°C. Aliquots (0.5 ml) of the supernatants containing the released ^32^P_i_ were transferred to scintillation vials containing 9.0 ml scintillation fluid.

### 9. Statistical analysis

All of the experiments were performed in triplicate, and similar results were obtained from at least three separate cell suspensions. The data were analyzed statistically using Student's t-test or one-way ANOVA followed by Tukey's test using the Prism computer software (Graphpad Software Inc., San Diego, CA, USA). A result was considered to be statistically significant when *p*<0.05.

## Results

**Figure 1:**
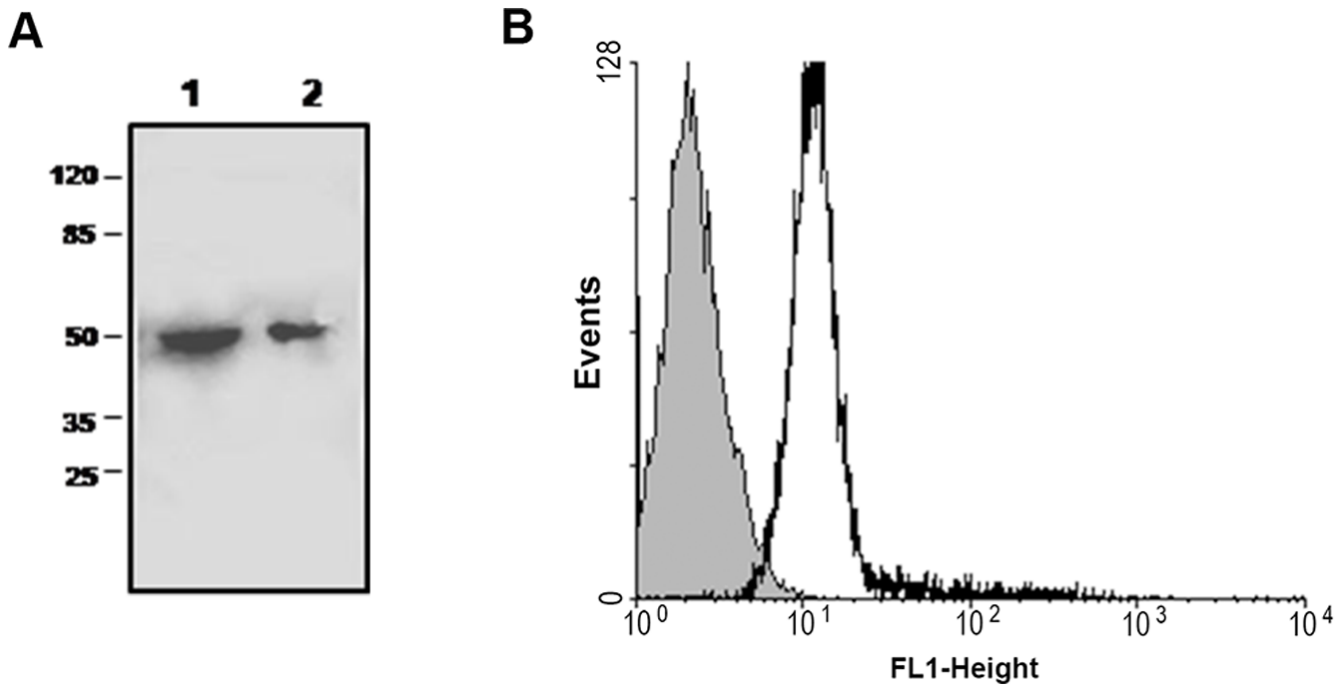
Western blot and flow cytometry analysis of *T. rangeli* incubated with the anti-326 peptide sequence of the AVP2 antibody. Panel A: *T. rangeli* and *T. cruzi* Dm 28c epimastigote proteins (50 µg/lane) were separated using 8% SDS-polyacrylamide gel electrophoresis and transferred to nitrocellulose. *Lane 1*, immunoblot probed with antiserum against H^+^PPase (AVP2) in the total extract of *T. rangeli*. The H^+^PPase antibody recognized a polypeptide with an apparent molecular mass of 64 kDa. *Lane 2*, immunoblot probed against the total extract of *T. cruzi*. Panel B: The intact cells of *T. rangeli* (5×10^6^ cells) were fixed in paraformaldehyde and sodium cacodylate buffer and stained with the anti-326 peptide sequence of *A. thaliana* vacuolar H^+^PPase (AVP2) produced in rabbit and an Alexa 488-conjugated anti-rabbit secondary antibody produced in mouse, with an emission in the range of 488 nm (red). Abscissa: fluorescence intensity; Ordinate: Events/Positive cells. The gray color represents the autofluorescence of the cells (without antibody).

**Figure 2:**
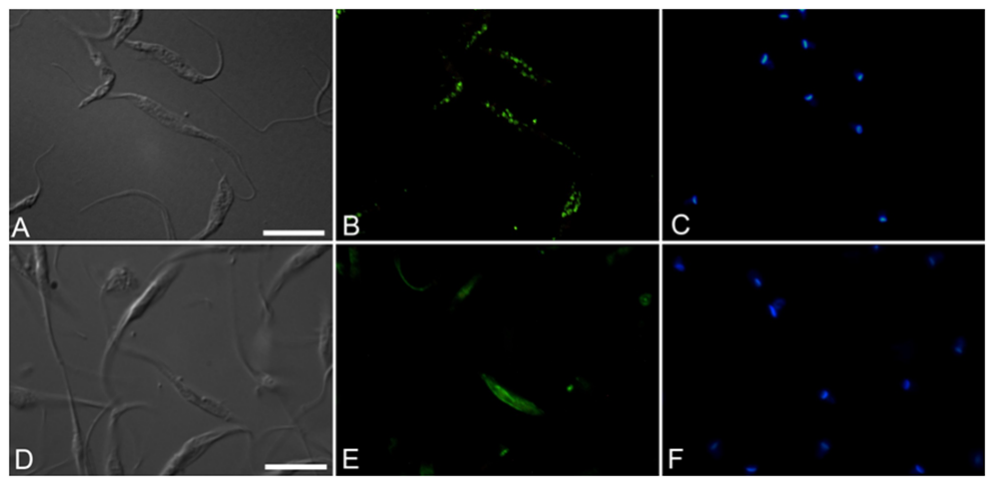
Immunolocalization of *T. rangeli* H^+^PPase using the anti-326 peptide sequence of the AVP2 antibody. Upper panels: cells permeabilized with Triton X-100; bottom panels: non-permeabilized cells, panels A and D: interferential differential contrast microscopy (DIC), panels B and E: immunofluorescence using anti-326 peptide sequence of AVP2 and Alexa 488; panels C and F: labeling of nuclear DNA with DAPI (2,6-diamino phenylindole). Bars: 10 µm.

The presence of a cDNA sequence amplified by the primers designed for the sequence of the H^+^-PPase of *T. cruzi* was detected in two different inocula of *T. rangeli*. This sequence was named *TrHPPase-1* (**Figure S1**). The PCR product was purified and sequenced. The obtained sequence has 98% identity to the *T. cruzi* vacuolar-type proton translocating pyrophosphatase 1 in the BLASTN analysis (data not shown). This result confirmed that the designed primers amplified the correct gene. Following the identification of a putative H^+^-PPase in *T. rangeli*, we searched for the recognition of this protein by an antibody directed against a 326 peptide sequence of *A. thaliana* AVP2. This antibody recognized a polypeptide with an apparent molecular mass of 64 kDa in the total extracts of the *T. cruzi* and *T. rangeli* epimastigotes (**Figure 1** **, panel A**). This protein was also recognized in the intact *T. rangeli* cells, as demonstrated by the rightward shift of the flow cytometry histogram (**Figure 1** **, panel B**). Using immunofluorescence microscopy, the cells permeabilized with Triton X-100 for 15 min showed positive staining for the putative H^+^-PPase in the intracellular organelles (presumably the acidocalcisomes) dispersed throughout the cytoplasm (**Figure 2**, **panel B**). However, when the cells were processed without the additional permeabilization step, the positive staining was mostly restricted to the plasma membrane of the parasites (**Figure 2**, **panel E**).

**Figure 3:**
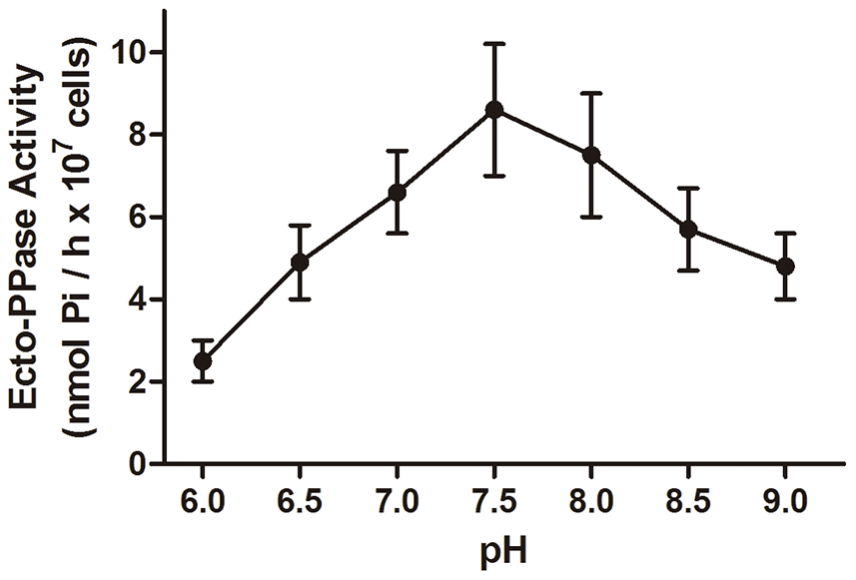
Effect of the pH of the reaction medium on the ecto-PPase activity of *T. rangeli*. Intact *T. rangeli* cells (10^7^ cells) were incubated in a reaction medium containing a buffer solution of 100 mM MES - HEPES - Tris with the pH adjusted from pH 6.0 to pH 9.0 with the addition of HCl and using 1 mM PPi as a substrate. The data represent the mean ± standard error using at least three different cell suspensions.

The intact *T. rangeli* cells did not hydrolyze the NPP substrates *p*-nitrophenyl-thymidine 5′-monophosphate (*p*-NP-5′-TMP) or *p*-nitrophenylphosphorylcholine (*p*-NPPC) (data not shown). For the PPi hydrolysis, **Figure 3** shows that the optimum pH for the ecto-PPase activity is 7.5, which is close to neutral.

Several ecto-enzymatic activities involved in the hydrolysis of phosphorylated substrates have been described in *T. rangeli*, including a Mg^2+^-dependent ecto-ATPase activity [57], [58] and an ecto-phosphatase activity capable of hydrolyzing β-GP [50], [59]. **Table 1** shows the influence of several inhibitors on these activities. The ecto-ATPase activity was not sensitive to any of the inhibitors tested, whereas the ecto-phosphatase activity was inhibited only by levamisole. The ecto-PPase activity was inhibited by NaF and AMDP, which were unable to exert any inhibitory influence on either the ecto-phosphatase or ecto-ATPase activities (**Table 1**).

**Table 1:** Effect of inhibitors on the ecto-ATPase, ecto-phosphatase and ecto-PPase activities of the intact cells of *T. rangeli*.

Additions	Ecto-ATPase Activity (%)	Ecto-phosphatase Activity (%)	Ecto-PPase Activity (%)
Levamizole, 1 mM	98.8±10.8	49.1±5.6*	122.1±7.5
Tartrate, 10 mM	117.0±8.4	106.5±9.7	96.4±3.5
Molybdate, 1 mM	103.6±12.7	95.8±8.6	113.3±0.8
Vanadate, 1 mM	91.5±5.4	99.2±10.9	131.4±11.7
NaF, 10 mM	107.8±18.3	104.9±13.4	25.9±0.6*
AMDP, 1 mM	124.7±2.1	86.5±16.6	12.3±1.2*

Intact *T. rangeli* cells (10^7^ cells) were used to measure each of the ecto-enzymatic activities in accordance with sections 7 and 8 (Materials and Methods). The activities are expressed as a percentage of that measured under the control conditions, i.e., without other additions. The data represent the mean ± standard error, using at least three different cell suspensions. *Denotes significant inhibition compared with the enzymatic activities of the control (no inhibitor).

**Figure 4:**
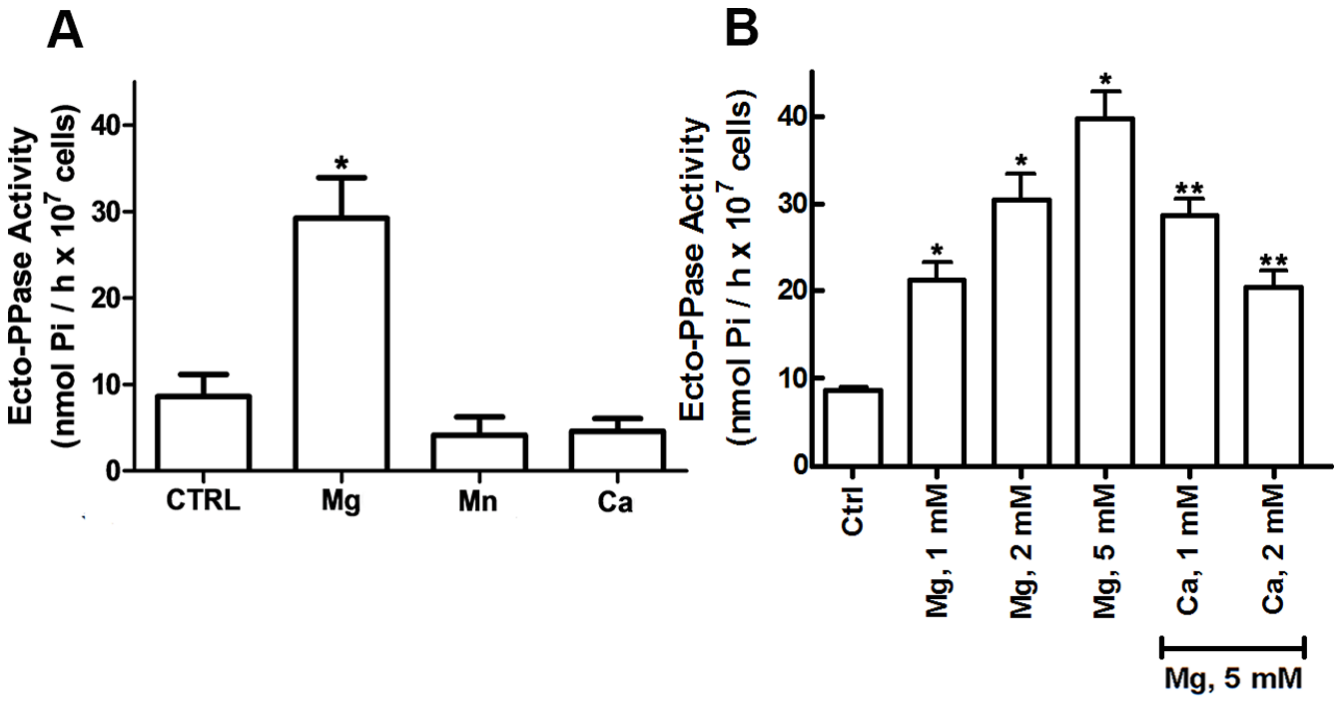
Effect of the divalent metals on the ecto-PPase activity of *T. rangeli*. Panel A: Intact *T. rangeli* cells (10^7^ cells) were incubated in a reaction medium containing a cold buffer solution of 50 mM Tris-HCl, pH 7.2, 100 mM sucrose and 20 mM KCl, using 1 mM PPi as substrate in the absence (control) or presence of MgCl_2_, MnCl_2_ or CalCl_2_ in a final concentration of 2 mM. Panel B: Intact *T. rangeli* cells (10^7^ cells) were incubated in the reaction medium described above, using 1 mM PPi as a substrate in the absence of a metal (control), in the presence of increasing concentrations of MgCl_2_ or in the presence of 5 mM MgCl_2_ and increasing concentrations of CaCl_2_. The data represent the mean ± standard error, using at least three different cell suspensions. *Denotes a statistically significant difference (*p*<0.05) compared with the control (no addition). **Denotes a significant difference (*p*<0.05) compared with the ecto-PPase activity in the presence of 5 mM MgCl_2_.

Soluble and H^+^-PPases are classified according to their sensitivity to Mg^2+^, Mn^2+^ and Ca^2+^. To determine whether these metals could modulate the ecto-PPase activity of *T. rangeli*, hydrolysis of PPi was measured in the presence of 2 mM CaCl_2_, MgCl_2_ or MnCl_2_. No modulatory effect was observed with the addition of MnCl_2_; however, the addition of MgCl_2_ was able to significantly stimulate this activity (**Figure 4**, **panel A**), and this stimulation occurred in a dose-dependent manner (**Figure 4**, **panel B**). The addition of CaCl_2_ inhibited, in a dose-dependent manner, the ecto-PPase activity stimulated by MgCl_2_ (**Figure 4**, **panel B**) but had no effect on the ecto-PPase activity in the absence of MgCl_2_ (**Figure 4**, **panel A**).

**Figure 5:**
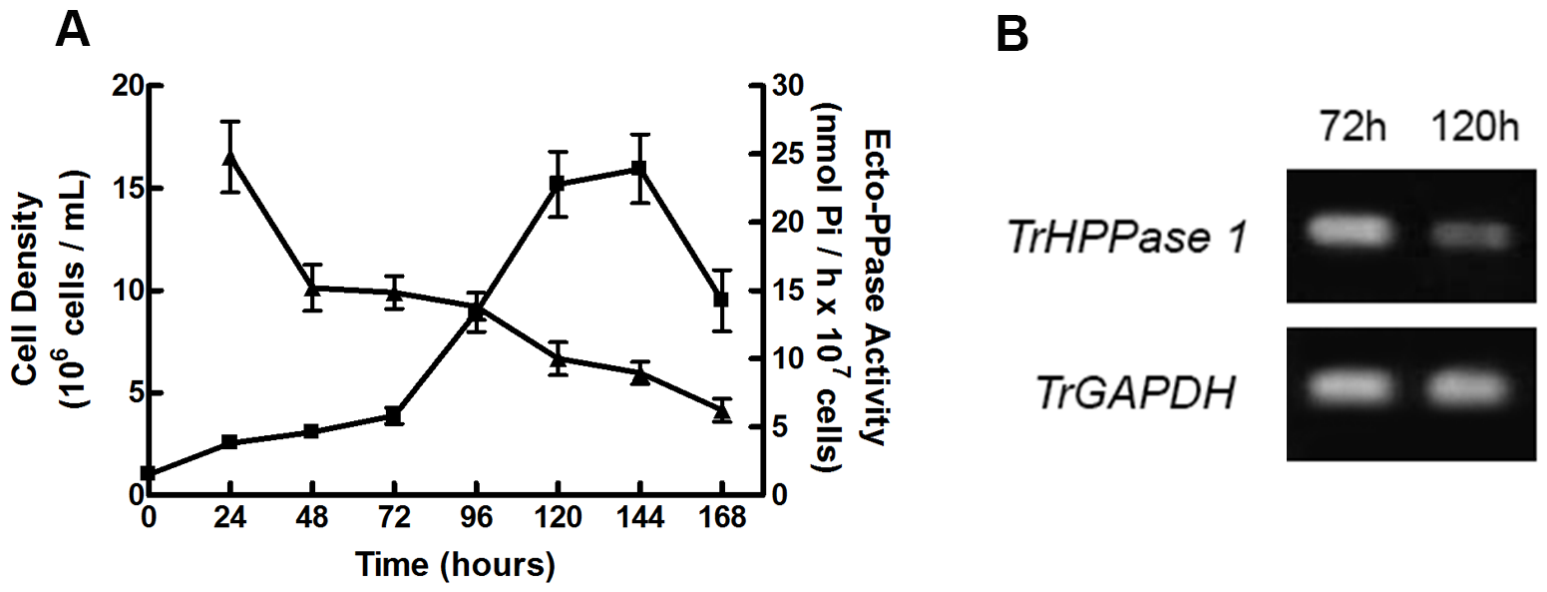
Ecto-PPase activity and gene expression along the *T. rangeli* proliferation. Panel A: Intact *T. rangeli* cells (10^6^ cells/ml) were inoculated in LIT culture, and the cell density was estimated daily by counting aliquots in a Neubauer chamber hemocytometer (squares); intact cells of *T. rangeli* (10^7^ cells) collected daily were incubated in a cold buffer solution of 50 mM Tris-HCl, pH 7.2, 100 mM sucrose and 20 mM KCl, using 1 mM PPi as a substrate. The data represent the average of an experiment performed in triplicate (triangles). Panel B: *T. rangeli* cells (10^8^ cells) were homogenized in TRIzol, and the total RNA was extracted. RNA samples were used to synthesize the complementary DNA (cDNA), and RT-PCR reactions were performed. The PCR products were subjected to agarose gel electrophoresis and visualized with UV light. Lines: 72 h and 120 h represent the time of parasite growth. *TrGAPDH* amplification was used as the positive control.

**Figure 6:**
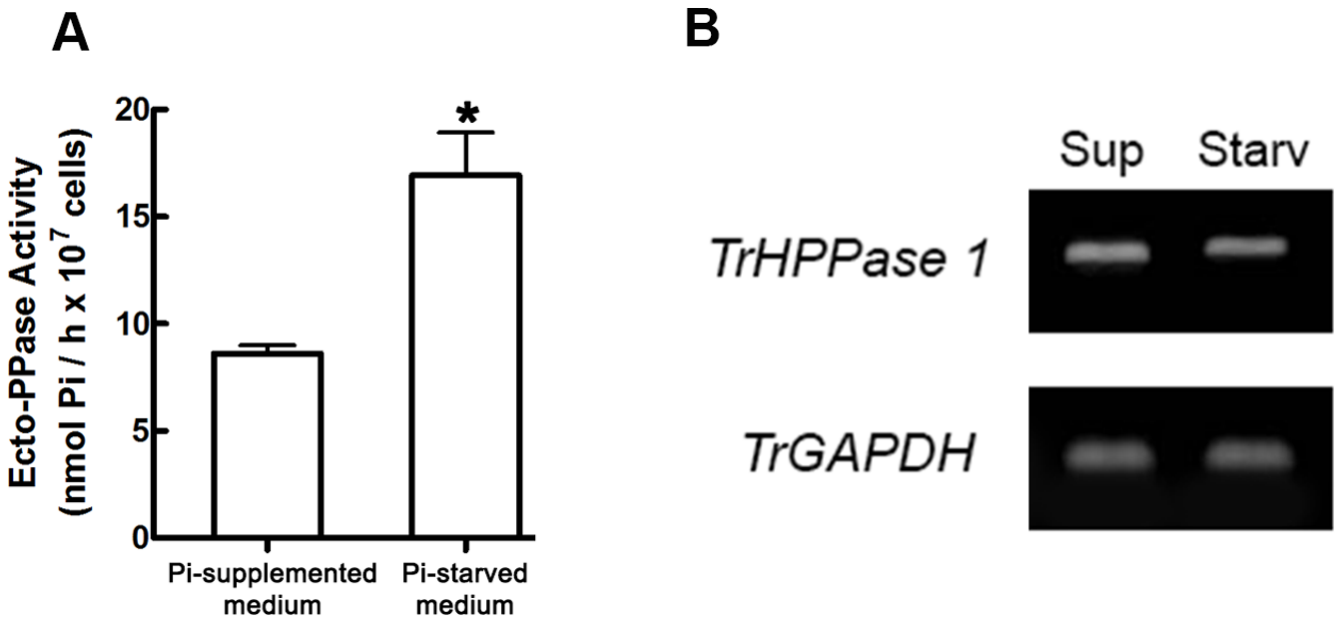
Ecto-PPase activity and gene expression in parasites submitted to different exogenous Pi content. Panel A: Intact *T. rangeli* cells (10^7^ cells) collected from a Pi-supplemented or a Pi-starved culture media were incubated in a cold buffer solution of 50 mM Tris-HCl, pH 7.2, 100 mM sucrose and 20 mM KCl, using 1 mM PPi as a substrate. The data represent the mean ± standard error, using at least three different cell suspensions. *Denotes a statistically significant difference (*p*<0.05). Panel B: *T. rangeli* cells (10^8^ cells) were homogenized in TRIzol, and the total RNA was extracted. RNA samples were used to synthesize the complementary DNA (cDNA), and RT-PCR reactions were performed. The PCR products were subjected to agarose gel electrophoresis and visualized with UV light. Lines: Sup represents parasites collected from Pi-supplemented culture media, and Starv represents parasites collected from Pi-starved culture media. *TrGAPDH* amplification was used as the positive control.

The ecto-PPase activity of *T. rangeli* decreased with increasing cell proliferation of the parasite (**Figure 5**, **panel A**). As observed in **Figure 5**, **panel B**, there is also a decrease in the expression of *TrHPPase* compared at 72 and 120 hours of parasite growth. Furthermore, the ecto-PPase activity from the cells grown in the Pi-starved medium was two times higher than that of the cells grown in the Pi-supplemented medium (**Figure 6**, **panel A**). However, the expression of *TrHPPase* did not change significantly in either of the conditions (**Figure 6**, **panel B**).

## Discussion

The first results in this study were generated through the sequence analysis of a gene in *A. thaliana* responsible for encoding AVP2, a vacuolar H^+^-PPase in this plant. Originally described as present only in vacuoles, this sequence presents a significant likelihood of being inserted into the plasma membrane. Additionally, by performing a search (BLAST) using the 326 peptide sequence of *A. thaliana* as the query sequence, we identified a protein sequence that matched with a high degree of alignment to many of the protozoa of the Trypanosomatidae family, such as *T. brucei*, *T. cruzi*, *Leishmania infantum*, *L. major* and *Leishmania braziliensis. T. cruzi* is evolutionarily close to *T. rangeli*, whose genome has not yet been elucidated. Furthermore, the H^+^-PPase of *T. cruzi* has a significant probability of being located in the plasma membrane [46]. Therefore, the gene sequence of the *T. cruzi* H^+^-PPase was used as a template to design a pair of primers to locate a coding sequence for a putative H^+^-PPase in *T. rangeli* using PCR. Additionally, we could use this approach to detect the expression of a putative H^+^-PPase in this parasite (**Figure S1**).

Recently, considerable advances have been made in the study of the mode of action of PPases. These enzymes can be isolated from all organisms because they are closely related to the synthesis of vital biopolymers. The primary structures of more than three dozen PPases have been determined, and this number is rapidly increasing with the successful sequencing of a large number of genomes. Sequence homology can vary considerably; however, a dozen and a half amino acid residues exposed to the active site cavity are conserved [60]. All of the PPases function as homo-oligomers. Prokaryotic enzymes are hexamers with a subunit molecular mass of ∼20 kDa. Eukaryotic PPases are dimers with subunits of ∼30 kDa [61]. Based on this homology, we used an antibody raised against a 326 peptide sequence of AVP2. This antibody reacted with a *T. rangeli* polypeptide of approximately 64 kDa as previously described in *T. cruzi* [27] (**Figure 1** **, panel A**). Using a fluorescent secondary antibody (Alexa 488), we showed that this antibody can also recognize a protein in the intact cells of *T. rangeli* (**Figure 1** **, panel B**).

Immunoelectron microscopy and biochemical studies have provided evidence for the presence of polypeptides of H^+^-PPase in the plasma membrane of various plants [62] and in unicellular eukaryotes, such as *T. cruzi* and *L. donovani* [9], [47]. In *T. cruzi*, the authors have used plasma membrane vesicles but have not clarified the exact location of the enzyme. However, the authors have managed to show that the enzyme exhibits certain different characteristics compared with the acidocalcisome enzyme. Thus, the only reports of ecto-PPase activities have been performed with the archaea *Sulfolobus acidocaldarius* [49] and with the trypanosomatids *L. donovani and L. amazonensis* [9], [37]. To confirm that the *T. rangeli* protein recognized by the anti-326 peptide sequence of AVP2 is located in the plasma membrane, we performed immunofluorescence microscopy. Performing the experiments in the intact parasites, we observed that this antibody recognized a protein on the outer surface of the plasma membrane. When the parasites were permeabilized, this antibody could also recognize the enzyme present in the acidocalcisomes (**Figure 2**).

There is a class of ecto-enzymes known as NPPs that are capable of hydrolyzing triphosphorylated nucleosides and monophosphoryl nucleotides with the release of PPi, which is also a substrate for these enzymes. One method used to biochemically detect the presence of NPPs is through the hydrolysis of specific substrates conjugated to *p*-nitrophenyl, which is easily measured by reading the yellow product *p*-nitrophenolates after the addition of a strong base. The participation of these enzymes in the hydrolysis of extracellular PPi was discarded because the intact cells of *T. rangeli* were unable to hydrolyze the NPP substrates *p*-NP-5′-TMP and *p*-NPPC (data not shown).

At a neutral pH, the equilibrium between the hexamer and trimer forms of the PPases is completely biased toward the hexamer, which is the more active form. A decrease in pH to 5.0–5.5 or an increase in pH to 8.5–9.0 causes a rapid dissociation of the hexamers, and the activity of the PPases decreases [61]. Therefore, the next biochemical parameter evaluated in our study was the influence of the pH of the reaction medium. **Figure 3** shows that the optimum pH for the ecto-PPase activity is very close to neutral, as indicated by the majority of the study with this enzyme in terms of its catalytic site, thereby suggesting that the ecto-PPase of *T. rangeli* may present oligomeric forms with different activities in response to the environmental pH.

Pyrophosphatases can be classified according to their sensitivity to Mg^2+^ and Mn^2+^ [39]–[42]. As observed in **Figure 4** **, panel A**, the ecto-PPase activity of *T. rangeli* was stimulated by MgCl_2_ but not by MnCl_2_. These data indicate that the ecto-PPase of *T. rangeli* has a profile similar to the members of the family I sPPases. This result is consistent with that observed in the ecto-PPase activity of *L. amazonensis* [37].

The effect of Ca^2+^ has also been widely discussed by various groups. The V-ATPases are completely insensitive to this metal [63], [64], whereas the pyrophosphatases are commonly inhibited by the formation of an unproductive PPi complex with calcium [65] or even by the effect of the metal itself in the form of free Ca^2+^ [63], [64]. The addition of CaCl_2_ inhibited, in a dose-dependent manner, the *T. rangeli* ecto-PPase activity stimulated by MgCl_2_ (**Figure 4**, **panel B**). The mechanisms of this inhibition have not been completely elucidated. However, this inhibition may be due to the competition between Ca^2+^ and Mg^2+^. Ca^2+^ can compete with Mg^2+^ to form a complex with PPi; however, the Ca^2+^-PPi complex is not a substrate for the enzyme. This mechanism of inhibition has already been proposed for other PPases and appears to be shared by different organisms because of the similarity in the active site structure of the enzyme [61].

In addition to Ca^2+^, other compounds can act as pyrophosphatase inhibitors. Mitochondrial pyrophosphatases, for example, suffer a 95% inhibition at fluoride concentrations close to 0.5 mM [66], whereas the enzyme present in the vesicles of *Vigna radiata* retains 15% of its activity in 10 mM fluoride [67]. Fluoride remains widely used as an inhibitor of pyrophosphatases in several systems; however, over the last decade, various analogues of PPi have been described. These analogues, known as bisphosphonates, can be significantly more specific than fluoride [68]. In these compounds, the central oxygen pyrophosphate is replaced by a carbon atom that prevents the breakage of the phosphoester linkages [69].

Other ecto-enzymes have been detected and characterized on the outer surface of the plasma membrane of this parasite, including a Mg^2+^-dependent ecto-ATPase [57], [58] and an ecto-phosphatase that uses β-GP as a substrate [50], [59]. To rule out the participation of these ecto-enzymes in the hydrolysis of PPi, we determined the sensitivity of each activity to classical phosphatase and pyrophosphatase inhibitors. We verified that the ecto-ATPase activity was not sensitive to any of the inhibitors tested; the ecto-phosphatase activity was inhibited only by levamisole, whereas the ecto-PPase activity was inhibited by NaF and AMDP (**Table 1**). These results suggest that the other ecto-enzymes of *T. rangeli* did not contribute to the observed hydrolysis of PPi, thereby confirming the fact that this hydrolysis arises from a specific pyrophosphatase.

Although PPi is used as a source of energy and phosphorus in various phosphorylating reactions, these reactions most likely consume only a minor proportion of intracellular PPi. If pyrophosphatases were not continually present in the cells, then the PPi concentration would rapidly increase to a level that would inhibit growth [70]–[72]. In this context, the next step in the study was to investigate the possible role that this ecto-PPase has in the physiology of the parasite. Initially, we measured the ecto-PPase activity over the proliferation of *T. rangeli in vitro* and observed that the ecto-PPase activity decreased with increasing cell proliferation of the parasite (**Figure 5**, **panel A**). Similar results have been obtained with the ecto-PPase activity of *L. amazonensis* and the ecto-phosphatase activity of *T. rangeli* [37], [50], thereby suggesting that these parasites activate the ecto-enzymatic activities related to phosphate metabolism during the exponential phase of growth. We could also observe a decrease in the expression of *TrHPPase* by comparing the third and fifth days of growth (**Figure 5**, **panel B**), indicating that the decrease in the activity may be related to a transcriptional control.

The ecto-PPase activity from the cells grown in the Pi-starved medium was significantly higher than that of the cells grown in the Pi-supplemented medium (**Figure 6**, **panel A**). However, this increase in the activity may not be related to an increase in the *TrHPPase* expression as observed in **Figure 6**, **panel B**. Our group has previously shown that the epimastigotes of *T. rangeli* are extremely dependent on the concentration of the exogenous Pi regarding their proliferation *in vivo* and *in vitro* [50], [51]. In these studies, the ecto-phosphatase activities of the parasite were also modulated by the availability of Pi in the culture medium and by the luminal contents of the organs of the insect vector in which the parasite multiplies. Thus, one of the possible functions of *T. rangeli* ecto-PPase could be to generate the extracellular Pi required for the parasite to correctly complete its cycle of proliferation.

The ecto-PPase activities have been recently described in two *Leishmania* species [9], [37], suggesting the presence of a genuine pyrophosphatase in the plasma membrane of trypanosomatids. A detailed search for the presence of ecto-PPase in different species, genera and families may provide important information regarding the physiological importance and molecular evolution of this enzyme.

## Supporting Information

Figure S1 **Gene expression of a putative proton-pyrophosphatase in** ***T. rangeli*** **.** *T. rangeli* cells (10^8^ cells) were homogenized in TRIzol, and the total RNA was extracted. RNA samples were used to synthesize the complementary DNA (cDNA), and RT-PCR reactions were performed. The PCR products were subjected to agarose gel electrophoresis and visualized with UV light. The amplicon size was estimated using ImageMasterTotalLab v. 1.11. The molecular size ladder used was the 100 bp ladder from Invitrogen/Life Technologies. Negative control: RT-PCR performed using cDNA synthesis performed without reverse transcriptase as the sample. Blank: RT-PCR performed using milli-Q water as the sample.
(TIF)
