# Supplementary figures and images for: Identification and Characterization of an Ecto-Pyrophosphatase Activity in Intact Epimastigotes of Trypanosoma rangeli
Source: PLoS One. 2014 Sep 9;9(9):e106852. doi: 10.1371/journal.pone.0106852 (PMC4159237; doi:10.1371/journal.pone.0106852)

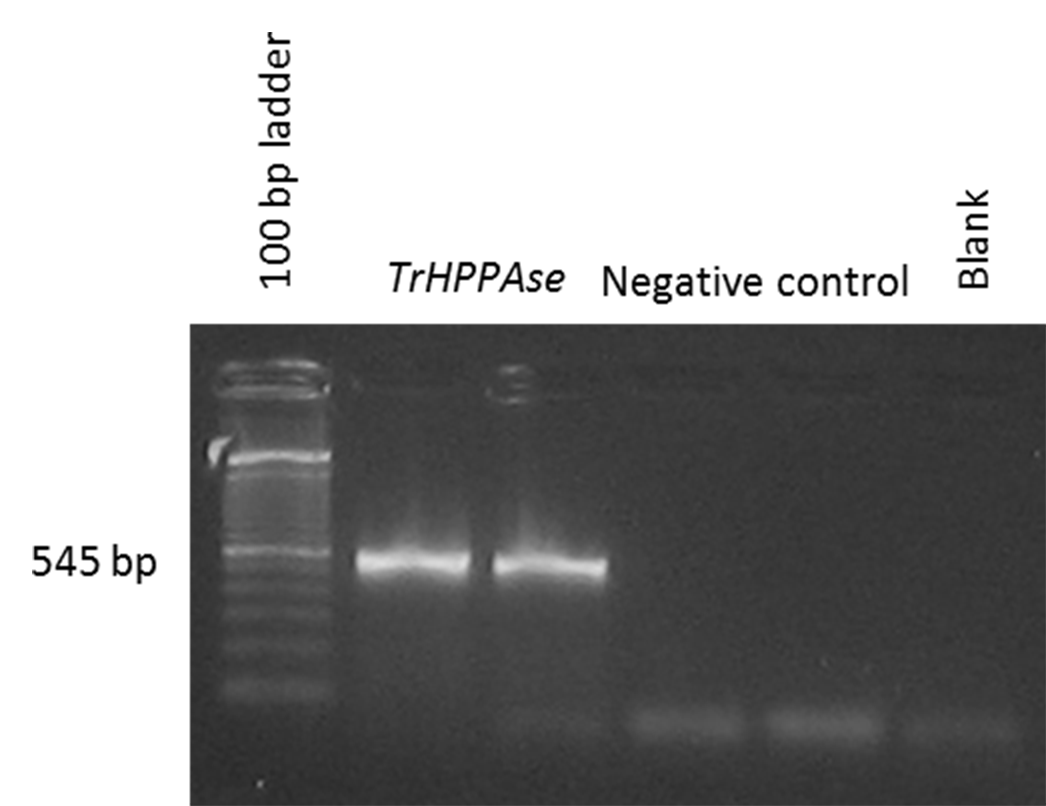

Supplement: Figure S1 — Gene expression of a putative proton-pyrophosphatase in T. rangeli . T. rangeli cells (108 cells) were homogenized in TRIzol, and the total RNA was extracted. RNA samples were used to synthesize the complementary DNA (cDNA), and RT-PCR reactions were performed. The PCR products were subjected to agarose gel electrophoresis and visualized with UV light. The amplicon size was estimated using ImageMasterTotalLab v. 1.11. The molecular size ladder used was the 100 bp ladder from Invitrogen/Life Technologies. Negative control: RT-PCR performed using cDNA synthesis performed without reverse transcriptase as the sample. Blank: RT-PCR performed using milli-Q water as the sample. (TIF) [file pone.0106852.s001.tif]
